# Supplementary material for: Electroseparation of Slaughterhouse By-Product: Antimicrobial Peptide Enrichment by pH Modification
Source: Membranes (Basel). 2020 May 3;10(5):90. doi: 10.3390/membranes10050090 (PMC7281006; doi:10.3390/membranes10050090)
Supplement: Supplementary file 1 [file membranes-10-00090-s001.zip › Supplementary Table S1.docx]

**Supplementary Table S1:** Identified peptide sequences into the recovery compartment

*These data were calculated with the available softwares on Expasy.org.*

| **Position** | **Sequence** | **MM** | **pI** | **Index** | **Whole** | **Without pH control** | **pH control (1%, w/v)** | | |
| --- | --- | --- | --- | --- | --- | --- | --- | --- | --- |
|  |  |  |  | **Gravy** | **hydrolysate** | **(1%, w/v)** | **4.7** | **6.5** | **9** |
| α74-82 | DDLPGALSE | 915 | 0.59 | -0.433 | + |  |  |  |  |
| α81-86 | SELSDL | 662 | 0.63 | -0.167 | + |  |  |  |  |
| β41-47 | FESFGDL | 813 | 0.63 | 0.171 | + |  |  |  |  |
| β45-51 | GDLSTAD | 677 | 0.73 | -0.471 | + |  |  |  |  |
| β1-6 | MLTAEE | 692 | 0.85 | -0.033 | + |  |  |  |  |
| α22-26 | AEYGA | 509 | 0.91 | -0.320 | + |  |  |  |  |
| β85-90 | AALSEL | 602 | 0.92 | 1.150 | + |  |  |  |  |
| α24-29 | YGAEAL | 622 | 0.92 | 0.367 | + |  |  |  |  |
| β85-89 | AALSE | 489 | 1.00 | 0.620 | + |  |  |  |  |
| α79-84 | ALSELS | 618 | 1.01 | 0.717 | + |  |  |  |  |
| α70-83 | VEHLDDLPGALSEL | 1507 | 3.42 | 0.086 | + |  |  |  |  |
| α72-82 | HLDDLPGALSE | 1166 | 3.54 | -0.300 | + |  |  |  |  |
| α129-135 | LANVSTV | 702 | 3.65 | 1.286 | + |  |  |  |  |
| α130-136 | ANVSTVL | 702 | 3.70 | 1.286 | + |  |  |  |  |
| α27-29 | EAL | 332 | 3.81 | 0.700 | + |  | + |  |  |
| α67-83 | TKAVEHLDDLPGALSEL | 1807 | 3.93 | -0.094 | + |  |  |  |  |
| α66-83 | LTKAVEHLDDLPGALSEL | 1920 | 3.93 | 0.122 | + |  |  |  |  |
| α64-83 | AALTKAVEHLDDLPGALSEL | 2062 | 3.93 | 0.290 | + |  |  |  |  |
| β3-13 | TAEEKAAVTAF | 1137 | 4.15 | 0.173 | + |  |  |  |  |
| β1-11 | MLTAEEKAAVT | 1163 | 4.15 | 0.273 | + |  |  |  |  |
| β1-12 | MLTAEEKAAVTA | 1234 | 4.15 | 0.400 | + |  |  |  |  |
| β2-13 | LTAEEKAAVTAF | 1250 | 4.15 | 0.475 | + |  |  |  |  |
| β1-13 | MLTAEEKAAVTAF | 1381 | 4.15 | 0.585 | + |  |  |  |  |
| β14-27 | WGKVKVDEVGGEAL | 1486 | 4.32 | -0.157 | + |  |  |  |  |
| β70-79 | FSNGMKHLDD | 1162 | 5.04 | -1.030 | + |  | + |  |  |
| β96-109 | HVDPENFKLLGNVL | 1594 | 5.17 | -0.036 | + |  |  |  |  |
| α60-80 | AKVAAALTKAVEHLDDLPGAL | 2102 | 5.23 | 0.486 | + |  | + |  |  |
| β82-84 | GTF | 323 | 5.50 | 0.567 | + |  | + |  |  |
| α95-97 | PVN | 328 | 5.50 | -0.300 | + |  | + |  |  |
| α41-43 | TYF | 429 | 5.50 | 0.267 | + | + | + |  |  |
| β9-13 | AVTAF | 507 | 5.50 | 1.980 | + |  | + |  |  |
| α129-134 | LANVST | 603 | 5.50 | 0.800 | + | + | + |  |  |
| β32-36 | VVYPW | 663 | 5.50 | 0.920 | + |  | + |  |  |
| α129-136 | LANVSTVL | 815 | 5.50 | 1.600 | + |  | + |  |  |
| β92-95 | CDKL | 478 | 5.92 | -2.925 | + |  | + |  |  |
| α92-98 | RVDPVNF | 845 | 6.25 | -0.271 | + |  | + | + |  |
| α110-122 | ASHLPSDFTPAVH | 1378 | 6.48 | -0.077 | + |  |  |  |  |
| α110-125 | ASHLPSDFTPAVHASL | 1649 | 6.48 | 0.237 | + |  |  |  |  |
| α109-125 | LASHLPSDFTPAVHASL | 1762 | 6.48 | 0.447 | + |  |  |  |  |
| α107-125 | VTLASHLPSDFTPAVHASL | 1962 | 6.48 | 0.584 | + |  | + | + |  |
| α110-128 | ASHLPSDFTPAVHASLDKF | 2039 | 6.48 | -0.042 | + |  |  |  |  |
| α126-133 | DKFLANVS | 892 | 6.66 | 0.113 | + |  |  |  |  |
| α126-136 | DKFLANVSTVL | 1206 | 6.66 | 0.745 | + |  |  |  |  |
| α123-128 | ASLDKF | 679 | 6.69 | 0.033 | + |  |  |  |  |
| α29-31 | LER | 416 | 6.86 | -1.400 | + |  | + | + |  |
| β14-30 | WGKVKVDEVGGEALGRL | 1812 | 6.91 | -0.194 | + |  |  |  |  |
| α118-128 | TPAVHASLDKF | 1185 | 7.50 | 0.064 | + |  |  |  |  |
| α102-106 | SHSLL | 555 | 7.54 | 0.560 | + |  | + | + |  |
| β70-84 | FSNGMKHLDDLKGTF | 1709 | 7.55 | -0.58 | + |  |  |  |  |
| α122-127 | HASLDK | 668 | 7.56 | -0.967 | + | + | + | + |  |
| α117-128 | FTPAVHASLDKF | 1185 | 7.56 | 0.292 | + |  | + | + |  |
| α84-98 | SDLHAHKLRVDPVNF | 1747 | 7.70 | -0.473 | + |  |  |  |  |
| α66-73 | LTKAVEHL | 910 | 7.80 | 0.287 | + |  |  |  |  |
| α101-106 | LSHSLL | 668 | 7.81 | 1.100 | + |  |  |  |  |
| α100-106 | LLSHSLL | 781 | 7.81 | 1.486 | + |  |  |  |  |
| α83-98 | LSDLHAHKLRVDPVNF | 1860 | 7.95 | -0.206 | + |  |  |  |  |
| α119-128 | PAVHASLDKF | 1084 | 8.15 | 0.140 | + |  |  |  |  |
| α119-125 | PAVHASL | 693 | 8.26 | 0.857 | + |  |  |  |  |
| β102-109 | FKLLGNVL | 903 | 8.88 | 1.325 | + |  | + |  |  |
| β114-125 | ARNFGKEFTPVL | 1378 | 8.88 | -0.225 | + |  |  |  |  |
| α36-46 | FPTTKTYFPHF | 1385 | 8.88 | -0.482 | + |  |  |  |  |
| α33-46 | FLSFPTTKTYFPHF | 1732 | 8.88 | 0.036 | + |  | + | + |  |
| β128-145 | DFQKVVAGVANALAHRYH | 1995 | 8.88 | -0.033 | + |  | + |  |  |
| α98-106 | FKLLSHSLL | 1057 | 9.06 | 1.033 | + |  |  |  |  |
| β138-145 | NALAHRYH | 980 | 9.41 | -1.038 | + |  |  |  |  |
| β126-145 | QADFQKVVAGVANALAHRYH | 2194 | 9.47 | -0.115 | + |  |  |  |  |
| α35-46 | SFPTTKTYFPHF | 1472 | 9.50 | -0.508 | + |  |  |  |  |
| β34-40 | YPWTQRF | 996 | 9.57 | -1.386 | + |  |  |  |  |
| α107-141 | VTLASHLPSDFTPAVHASLDKFLANVSTVLTSKYR | 3787 | 9.64 | 0.231 | + |  |  |  |  |
| α34-43 | LSFPTTKTYF | 1204 | 9.74 | -0.030 | + |  | + | + |  |
| α34-46 | LSFPTTKTYFPHF | 1585 | 9.74 | -0.177 | + |  | + | + |  |
| β32-40 | VVYPWTQRF | 1195 | 9.81 | -0.144 | + | + | + | + | + |
| β130-139 | QKVVAGVANA | 956 | 9.83 | 0.670 | + | + | + | + |  |
| β135-145 | GVANALAHRYH | 1208 | 9.83 | -0.245 | + |  |  |  |  |
| β31-40 | LVVYPWTQRF | 1308 | 9.83 | 0.250 | + | + | + | + | + |
| α54-59 | QVKGHG | 624 | 9.84 | -1.200 | + |  | + |  |  |
| α87-98 | HAHKLRVDPVNF | 1432 | 9.87 | -0.550 | + |  |  |  |  |
| α58-64 | HGAKVAA | 652 | 9.88 | 0.300 | + |  | + |  |  |
| β129-137 | FQKVVAGVA | 918 | 9.88 | 1.244 | + |  |  |  |  |
| β137-145 | ANALAHRYH | 1052 | 9.90 | -0.722 | + |  |  |  |  |
| α99-104 | KLLSHS | 683 | 9.91 | -0.183 | + |  |  |  |  |
| β7-13 | KAAVTAF | 706 | 9.91 | 1.114 | + |  |  |  |  |
| β103-109 | KLLGNVL | 755 | 9.91 | 1.114 | + | + | + | + |  |
| α99-105 | KLLSHSL | 796 | 9.91 | 0.386 | + | + | + | + |  |
| β103-110 | KLLGNVLV | 855 | 9.91 | 1.500 | + |  |  |  |  |
| β7-14 | KAAVTAFW | 893 | 9.91 | 0.863 | + |  |  |  |  |
| α99-106 | KLLSHSLL | 910 | 9.91 | 0.812 | + | + | + | + | + |
| α99-107 | KLLSHSLLV | 1009 | 9.91 | 1.189 | + |  | + |  |  |
| α37-46 | PTTKTYFPHF | 1238 | 10.09 | -0.810 | + | + | + | + |  |
| β114-124 | ARNFGKEFTPV | 1247 | 10.18 | -0.591 | + | + | + | + | + |
| α134-141 | TVLTSKYR | 967 | 10.40 | -0.487 | + | + | + | + |  |
| β130-145 | QKVVAGVANALAHRYH | 1733 | 10.40 | 0.006 | + |  |  |  |  |
| α133-141 | STVLTSKYR | 1054 | 10.41 | -0.522 | + |  |  |  |  |
| β129-135 | FQKVVAGVANALAHRYH | 1880 | 10.41 | 0.171 | + |  | + |  |  |
| α135-141 | VLTSKYR | 866 | 10.46 | -0.457 | + | + | + | + |  |
| α136-141 | LTSKYR | 766 | 10.47 | -1.233 | + | + | + | + |  |
| α129-141 | LANVSTVLTSKYR | 1451 | 10.47 | 0.123 | + | + | + | + | + |
| α137-141 | TSKYR | 653 | 10.50 | -2.240 | + | + | + | + | + |
| α37-40 | PTTK | 445 | 10.57 | -1.725 | + |  | + | + |  |
| α56-67 | KGHGAKVAAALT | 1153 | 10.69 | 0.225 | + |  |  |  |  |
| β35-40 | PWTQRF | 833 | 11.29 | -1.400 | + |  |  |  |  |
