# Supplementary material for: Electroseparation of Slaughterhouse By-Product: Antimicrobial Peptide Enrichment by pH Modification
Source: Membranes (Basel). 2020 May 3;10(5):90. doi: 10.3390/membranes10050090 (PMC7281006; doi:10.3390/membranes10050090)
Supplement: Supplementary file 1 [file membranes-10-00090-s001.zip › Supplementary Figure S1.docx]

**Supplementary Figure S1:** Evolution of KCl conductivities (**a, c**) and current intensities (**b, d**) during the EDUF treatment.

**
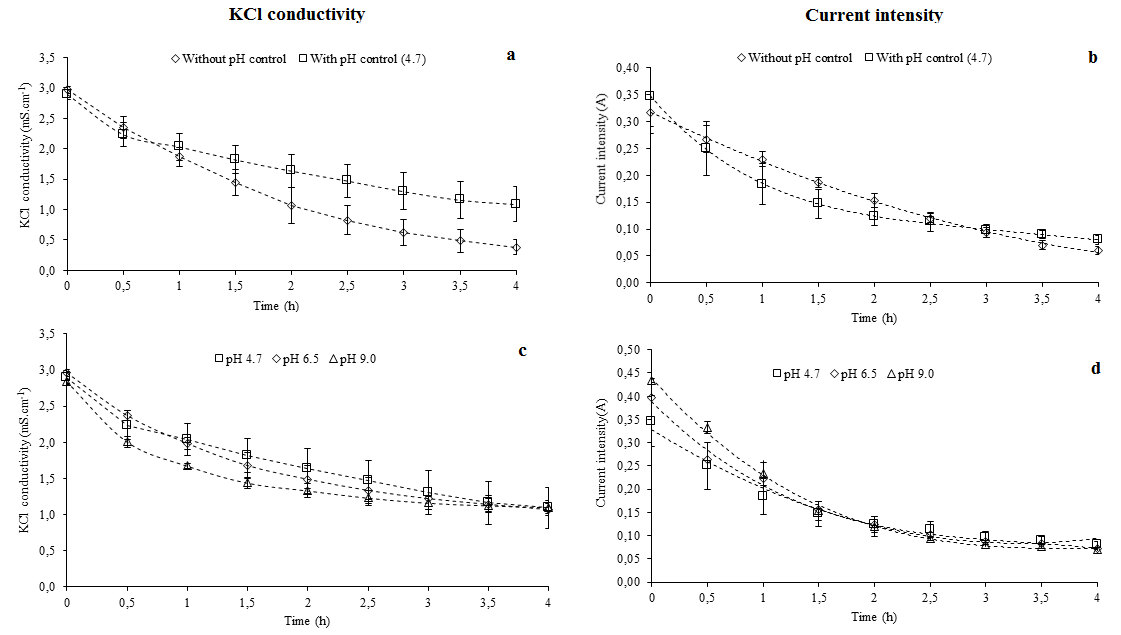
**
